# Measuring vascular reactivity with breath‐holds after stroke: A method to aid interpretation of group‐level BOLD signal changes in longitudinal fMRI studies

**DOI:** 10.1002/hbm.22735

**Published:** 2015-02-27

**Authors:** Fatemeh Geranmayeh, Richard J.S. Wise, Robert Leech, Kevin Murphy

**Affiliations:** ^1^ Computational Cognitive and Clinical Neuroimaging Laboratory Imperial College, Hammersmith Hospital London W12 0NN United Kingdom; ^2^ CUBRIC School of Psychology, Cardiff University Cardiff CF10 3AT United Kingdom

**Keywords:** vascular reactivity, stroke, fMRI, breath‐hold

## Abstract

Blood oxygenation level‐dependent (BOLD) contrast functional magnetic resonance imaging (fMRI) is a widely used technique to map brain function, and to monitor its recovery after stroke. Since stroke has a vascular etiology, the neurovascular coupling between cerebral blood flow and neural activity may be altered, resulting in uncertainties when interpreting longitudinal BOLD signal changes. The purpose of this study was to demonstrate the feasibility of using a recently validated breath‐hold task in patients with stroke, both to assess group level changes in cerebrovascular reactivity (CVR) and to determine if alterations in regional CVR over time will adversely affect interpretation of task‐related BOLD signal changes. Three methods of analyzing the breath‐hold data were evaluated. The CVR measures were compared over healthy tissue, infarcted tissue and the peri‐infarct tissue, both sub‐acutely (∼2 weeks) and chronically (∼4 months). In this cohort, a lack of CVR differences in healthy tissue between the patients and controls indicates that any group level BOLD signal change observed in these regions over time is unlikely to be related to vascular alterations. CVR was reduced in the peri‐infarct tissue but remained unchanged over time. Therefore, although a lack of activation in this region compared with the controls may be confounded by a reduced CVR, longitudinal group‐level BOLD changes may be more confidently attributed to neural activity changes in this cohort. By including this breath‐hold‐based CVR assessment protocol in future studies of stroke recovery, researchers can be more assured that longitudinal changes in BOLD signal reflect true alterations in neural activity. *Hum Brain Mapp 36:1755–1771, 2015*. © **2015 The Authors Human Brain Mapping Published by Wiley Periodicals, Inc**.

## INTRODUCTION

Over recent decades, functional magnetic resonance imaging (fMRI) has become a useful tool when studying the recovery of brain function following a stroke or other focal brain injury. Interpretations of fMRI findings should take into account the limitations of this technique, and factors that influence the fMRI‐derived blood oxygenation action level‐dependent (BOLD) signal [Logothetis, 2008]. BOLD contrast fMRI is not only dependent on increased oxygen metabolism due to increased neural activity since activation‐related differences also depend on changes in cerebral blood flow (CBF) and cerebral blood volume [Buxton, 2012; Davis et al., 1998; Hoge et al., 1999]. Since stroke has a vascular etiology, this may alter the normal neurovascular coupling between CBF and oxygen consumption (taken as representative of neural activity), with resulting uncertainty when interpreting BOLD signal changes.

Reductions in cerebrovascular reactivity (CVR), the response of cerebral blood vessels to a vasodilatory stimulus, are associated with factors that predispose to stroke [Gupta et al., 2012; Markus, 2001]. Acute vascular changes as a result of stroke may further reduce CVR. Reductions in CVR will lead to alterations in neurovascular coupling; that is, a given change in neural activity will no longer be represented by the same BOLD signal change. Regional changes in CVR and BOLD signal have been observed in adults after stroke [Krainik et al., 2005]. Combining BOLD fMRI with a vasoactive stimulus such as CO_2_ provides a robust means of characterizing CVR [Shiino et al., 2003]. In many studies of clinical populations, an increase in arterial CO_2_ levels is achieved using administration of a vasodilatory gas through a face mask during scanning [Heyn et al., 2010]. In patients with stroke, this may not always be desirable or feasible due to the discomfort of the experimental setup.

A simple method to measure BOLD CVR in patient groups using a breath‐hold task has been developed [Bright and Murphy, 2013; Murphy et al., 2011]. Breath‐holding offers an alternative to gas administration, and provides a similar BOLD CVR measure [Kastrup et al., 2001]. Until recently, it had been assumed that the degree of cooperation to perform a breath‐holding task would vary too greatly across a patient population to provide a reliable measure of CVR [Spano et al., 2013]. However, Bright and Murphy [2013] have shown that by monitoring end‐tidal CO_2_ levels during a breath‐hold task, robust and repeatable measures of CVR can be obtained, even if the breath‐hold task is poorly performed. This opens the way to using such a task in a stroke population to account for CVR differences. These measures can, in turn, be used to adjust the BOLD signal to more accurately reflect the neural responses to stimuli in a task‐related fMRI study [Murphy et al., 2011].

Changes in CVR over time may potentially be problematic, particularly in longitudinal fMRI studies investigating recovery after the onset of stroke [Saur, 2006; Ward et al., 2003]. In the longitudinal study by Saur et al.[2006], investigating language recovery following a left hemisphere stroke, there was no activity in the left hemisphere language areas, within the same vascular territory as the infarct, during the first few days after the stroke. Although this was interpreted as a lack of language‐related neural activity caused by the stroke, an alternative explanation is that the absence of BOLD signal was the result of altered cerebral haemodynamics rather than a decline in neural activity per se. For example, it has been shown that the morphology of the haemodynamic response function (HRF) in the left perisylvian area is altered in some aphasic patients following a stroke [Bonakdarpour et al., 2007]. In another study, the characteristics of the HRF response to an auditory comprehension task changed as patients progressed from the acute to the sub‐acute phase of stroke [Altamura et al., 2009]. In addition, major arterial stenoses result in a compensatory reduction in distal cerebrovascular resistance, which in turn will alter CVR. Thus, it has been demonstrated that the HRF ipsilateral to an internal carotid artery stenosis has different temporal characteristics to that in the contralateral hemisphere [Carusone and Srinivasan, 2002]. Furthermore, large vessel cervical or cerebral artery steno‐occlusive disease can induce competitive intra‐cerebral redistribution of flow from territories with low vasodilatory reserve to those with high reserve [Sobczyk et al., 2014]. Thus without taking into account acute and chronic changes in normal CVR, task‐related BOLD signal changes over time, particularly in longitudinal studies of stroke, are prone to misinterpretation.

One fMRI study investigating aphasic stroke recovery has used a simple breath‐hold task to account for differences in haemodynamic responsiveness in stroke patients [van Oers et al., 2010]. One advantage of using the Bright and Murphy [2013] approach, compared with the method used in that study, is that it quantifies the end‐tidal CO_2_ changes to the breath‐holds. This compensates for variability in task performance across the patients, providing more reliable quantification of the CVR. The end‐tidal CO_2_ measure provides an accurate model of the expected BOLD response for an individual patient, allowing for the different delays in the breath‐hold response across varying brain regions. In addition, the use of paced breathing between breath‐hold challenges in the Bright and Murphy [2013] method leads to a more consistent baseline condition, and thus a less variable measure [Scouten and Schwarzbauer, 2008]. Finally, the Bright and Murphy [2013] approach uses end‐expiration measures, avoiding the biphasic BOLD response that is observed in end‐inspiration challenges [Thomason and Glover, 2008]. This leads to a more repeatable starting point for the breath‐hold challenge, resulting in improved reproducibility [Scouten and Schwarzbauer, 2008].

The purpose of this study is to demonstrate the feasibility of this breath‐hold technique in patients with stroke, and to determine whether, in this cohort, alterations in CVR in the peri‐infarct tissue over time might result in misinterpretation of group‐level task‐related BOLD signal changes. Three methods of analyzing the data were investigated: *GlobOpt*, in which a single model of the BOLD response was derived from the end‐tidal CO_2_ trace; *VoxOpt*, when the delay between the end‐tidal CO_2_ and fMRI time series was allowed to vary on a voxel‐wise basis to account for slower regional haemodynamic responses; and *RHsig*, a method by which a BOLD model was derived from the contralesional right hemisphere and does not require measurement of the end‐tidal CO_2_. The CVR measures were compared in healthy, peri‐infarct tissue and infarcted tissue at two time points after the ictus, in the sub‐acute and chronic phase. The peri‐infarct tissue was specifically included in the analysis as some studies have attributed the level of BOLD signal change in this region to stroke recovery [Heiss et al., 1999, 2006; Rosen et al., 2000; Saur et al., 2006]. The results demonstrate the use of this method to assess CVR in a cohort of patients with stroke, and suggest that inclusion of this protocol in longitudinal BOLD studies of stroke recovery will allow more confident interpretation of group results.

## METHODS

### Participants

Forty‐six stroke patients (referred to as the PT group) with left lateralized cerebral infarction were scanned (30 male, average age 61.2 years, range 26–79 years). Table 1 shows their demographic data, including cerebrovascular risk factors, details of the size and site of the stroke lesion, the mode and outcome of vascular imaging and the timing of the fMRI scans. The majority of patients had embolic stroke that was either from a cardio‐embolic source (e.g. atrial fibrillation, or patent foramen ovale), or artery‐to‐artery embolism (e.g. from the carotid tree). There were 18 patients with occlusive arterial disease either in the intracranial vessels (e.g. due to stenosis or fresh thrombus) or dissection in the carotid tree.

**Table 1:** List of patients

Patient	Sex	Age (years)	SPPS scan (days post stroke)	CPPS scan (days post stroke)	Scan interval (days)	Cerebrovascular risk factors	Vascular stenosis	Imaging modality used for vascular imaging	Lesion Location	Lesion Size (cm^3^)
1	M	77	28	104	76	A, H	NSS	Carotid Doppler	C, SC wm (F, I)	23.4
2	F	46	14	119	105	eS	NSS	CTA intracranial and cervical vessels	C, SC wm (F, I)	2.8
3	F	77	11	144	133	S	NSS	Carotid Doppler	SC wm, SC gm (I)	1.2
4	M	50	12	102	90	H, Is, D, Cl	NSS	Carotid Doppler	C, SC wm (F, I O, P)	13.0
5	M	44	12	161	149	Cl, S	Underwent successful endarterectomy of 90% L ICA stenosis before the study. Stenosis of R vertebral artery with full distal reconstitution with collaterals	CTA intracranial and cervical vessels	SC wm, SC gm (F, I)	7.3
6	M	46	15	200	185	D, H, Cl	Short L M1 segment stenosis	CTA intracranial and cervical vessels	C, SC wm, SC gm (F, P, T, O and right F)	13.8
7	M	76	25	124	99	A, H	NSS	Carotid Doppler	C, SC wm (P, I)	0.3
8	M	60	10	127	117	D, H, Ti, Cl	NSS	Carotid Doppler	SC wm (F)	4.8
9	M	56	17	96	79	D, H	NSS	Carotid Doppler	C, SC wm (P, F, T)	14.1
10	M	57	20	90	70	S	Complete stenosis of L MCA	MRA intracranial and cervical vessels	C, SC wm (P, F)	34.3
11	M	75	16	101	85	Cl, Is	Asymptomatic L ICA 90% stenosis	CTA intracranial and cervical vessels	C, SC wm (T, O) posterior circulation	18.3
12	M	65	6	101	95	‐	NSS	Carotid Doppler	C, SC wm (F, I)	9.1
13	M	64	6	89	83	Cl	Full occlusion of L M3	CTA intracranial and cervical vessels	C, SC wm (I, F, P)	33.9
14	M	64	12	96	84	A, H, Cl	NSS	Carotid Doppler	C, SC wm (F, P)	10.5
15	F	39	20	91	71	Ti	NSS	CTA intracranial and cervical vessels	C, SC wm (F, I)	7.9
16	M	65	11	104	93	H, I, Cl, S	Asymptomatic L vertebral stenosis	CTA intracranial and cervical vessels	C, SC gm (T)	4.5
17	F	49	18	88	70	‐	NSS	MRA intracranial and cervical vessels	C (F)	1.4
18	M	53	5	102	97	‐	NSS	CTA intracranial and cervical vessels	C SC wm (F)	0.8
19	F	69	9	87	78	H, eS, D	NSS	Carotid Doppler	C, SC wm (T,P,I)	75.4
20	M	54	14	99	85	Is, H, Cl	NSS	Carotid Doppler	C, SC wm (F)	19.6
21	F	53	8			H, A	NSS	Carotid Doppler	C, SC wm (F)	16.7
22	M	63	7			Cl	NSS	Carotid Doppler	SC wm	2.6
23	M	50	20			D, H, Cl, S	NSS	Carotid Doppler	C, SC wm (I, T)	3.1
24	M	75	14			Cl, Is, D	NSS	Carotid Doppler	SC gm	1.4
25	M	67		90		eS, H, A, Is	L M1 thrombus	MRA intracranial and cervical vessels	C, SC wm (I, F, T, P)	49.1
26	M	79		93		Cl	NSS	Carotid Doppler	C, SC wm (T, F, P)	2.5
27	F	79		94		A, Cl	L M2 thrombus	CTA intracranial and cervical vessels	C, SC wm, SC gm (I, F)	6.9
28	M	79		118		A, Is, Cl, H	NSS	Carotid Doppler	C, SC wm (I F)	3.0
29	M	67		101		H, Cl	NSS	MRA intracranial and cervical vessels	C, SC gm, SC wm (F, O, P)	17.6
30	M	56		126		H, Is	NSS	Carotid Doppler	SC wm (F, P, T, O and right P, F)	12.6
31	M	75		84		H, S	NSS	Carotid Doppler	SC wm	0.5
32	F	30		100		‐	NSS	MRA intracranial and cervical vessels	C, SC gm, SC wm (I, F, T, P)	49.7
33	F	74		105		H, Cl	NSS	CTA intracranial and cervical vessels	SC wm (O, P)	5.1
34	F	68		91		‐	NSS	MRA intracranial and cervical vessels	SC wm	4.8
35	F	74		101		Cl	Moderate short M1 stenosis	CTA intracranial and cervical vessels	SC wm (F, O, T, P)	10.7
36	F	61		160		A, S	L M1 thrombus with full recanalization after thrombectomy	DSA + CTA intracranial and cervical vessels	SC wm, C (F, I, T, P)	168.0
37	M	66		109		eS, H	NSS	MRA intracranial and cervical vessels + Carotid Doppler	C (F)	7.5
38	M	63		111		A, D, Cl, H	50‐70% stenosis L CCA	MRA intra and extra cranial and Carotid Doppler.	C, SC wm, SC gm (F, P T)	31.3
39	M	68		189		‐	L ICA dissection	MRA intracranial and cervical vessels	SC wm, SC gm, C (I, F, P, T)	144.0
40	M	75		122		Is, Cl, H, D, eS	NSS	Carotid Doppler	C, SC wm, SC gm, (I, F, P)	82.0
41	F	54		98		S	L ICA aneurysm repair prior fMRI	CTA intracranial and cervical vessels	SC wm, SC gm (T, P, F, O)	33.7
42	F	26		170		Cl	L M1 thrombus with full recanalization after thrombectomy	DSA + CTA intracranial and cervical vessels	C, SC wm (F, I, P, T)	53.0
43	M	48		94		‐	L ICA dissection	CTA intracranial and cervical vessels	SC wm (I, F, T, P)	71.9
44	F	62		182		H, eS, Cl	L ICA dissection	CTA intracranial and cervical vessels	SC wm (F, P)	12.4
45	F	38		105		D, S, A, H, Cl	L ICA dissection	CTA intracranial and cervical vessels	C, SC wm, SC gm (I, F, P, O, T)	104.0
46	M	79		104		Is, Ti, H, Cl, S	Asymptomatic R ICA 70% stenosis	Carotid Doppler	C, SC wm (I, P, T, O)	43.9

Top, middle and bottom sections show the details of patients with BH scans at two time points, only at *SPPS*, and only at *CPPS*, respectively. All patients had strokes caused by cerebral infarction. All patients had carotid artery imaging to ascertain the degree of carotid stenosis. A proportion had additional vertebral artery or intracranial arterial imaging. In the context of this paper we have stated the degree of carotid tree stenosis if it was >50%, which is less than the threshold of 70% stenosis deemed to be clinically significant. A number of patients had significant carotid artery stenosis and the majority had multiple cerebrovascular risk factors, all of which could potentially impact the cerebrovascular reactivity.

L, left; R, right; ICA, internal carotid artery; CCA, common carotid artery; MCA, middle cerebral artery; CTA, CT angiography; MRA, MR angiography; DSA, digital subtraction angiography; NSS, no significant stenosis; M1‐3 refer to branches of left middle cerebral artery. H, hypertension; Cl, hypercholesterolemia; Is, ischaemic heart disease; Ti, previous small cerebrovascular disease or transient ischaemic attacks; S, smoker; eS, ex‐smoker; A; atrial fibrillation. Lesion location is in the left hemisphere unless stated otherwise: C, cortical; SC wm, subcortical white matter; SC gm, subcortical grey matter; I, insular; F, frontal; P; parietal, T; temporal; O, occipital.

**Figure 1:**
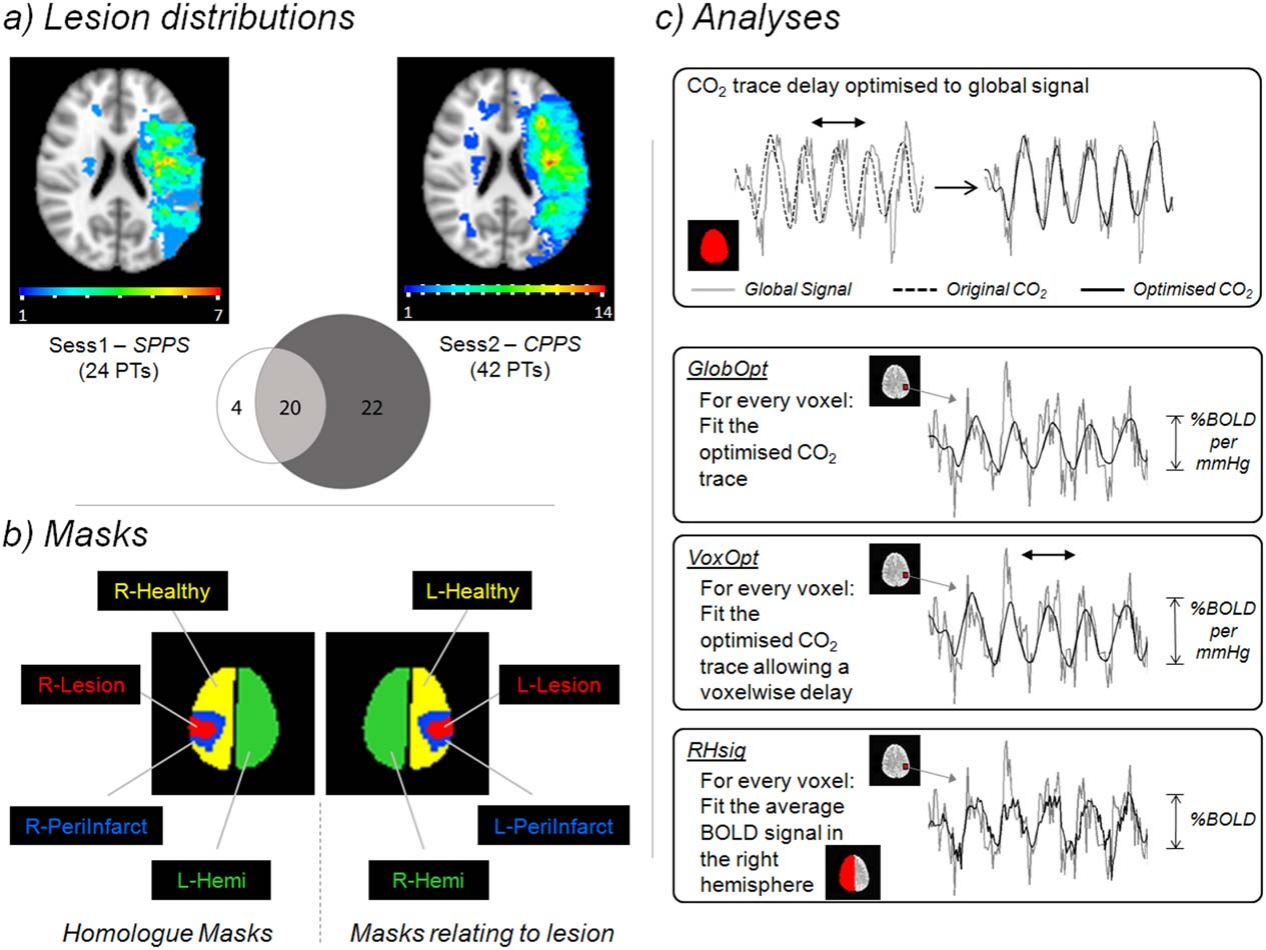
(**a**) Lesion Distributions. The spatial distribution of the patient's acute infarct lesions is shown for both the Sub‐acute Phase Post‐Stroke (*SPPS* also referred to as *Sess1*) and Chronic Phase Post‐Stroke (*CPPS* also referred to as *Sess2*) time points. The colour‐code of each voxel indicates how many patients had a lesion that included that voxel. The Venn diagram shows how many patients were scanned at each time point and the overlap between them. (**b**) Masks. The schematic shows the masks used in the analysis. (**c**) Analyses. The delay of each participant's end‐tidal CO_2_ trace was initially optimised by aligning it to the corresponding global BOLD signal time series. Three subsequent analyses types were performed. *GlobOpt*: the optimised CO_2_ trace was fitted to each voxel's time series using a GLM. *VoxOpt*: the same CO_2_ trace was fitted to the voxel's time series but was allowed to vary temporally to optimise the fit. *RHsig*: the average BOLD time series signal from the right hemisphere was fitted to the time series of every voxel in the brain. [Color figure can be viewed in the online issue, which is available at http://wileyonlinelibrary.com.]

Twenty‐four patients were scanned in the sub‐acute phase post‐stroke (*SPPS*, also referred to as *Sess1*) and 42 in the chronic phase post‐stroke (*CPPS*, also referred to as *Sess2*) (Fig. 1a). Mean time after stroke for *SPPS* was 14 days (range 5–28 days) and for *CPPS* was 114 days (range 84–200 days). Additionally, 26 healthy volunteers (referred to as the *HV* group) were scanned (9 male, average age 56.5 years, range 37–78 years), 17 of whom were scanned again after approximately 100 days. There was no between‐group differences in age (*t*‐test *P* = 0.15) or sex (Fisher's exact test *P* = 0.5). The National Research Ethics Service Committee (West London) approved the study.

### Scanning Parameters

MRI data were obtained on a Siemens Magnetom Trio 3 Tesla scanner. Whole brain, dual‐echo, BOLD GE‐echoplanar imaging (EPI) images (repetition time (TR) = 2 s, echo time (TE1) = 13 ms, TE2 = 31 ms, voxel‐size 3.5 × 3.5 × 3 mm^3^, 36 slices) were collected during a breath‐hold task (see below). Only the second echo was analysed in this study. Quadratic shim gradients were used to correct for magnetic field inhomogeneities within the brain, and residual field artefacts were compensated after acquiring fieldmap images. A high‐resolution 1 mm^3^ isotropic T_1_‐weighted whole‐brain structural image was obtained.

In addition, diagnostic diffusion‐weighted MRI (DWI) and apparent diffusion co‐efficient (ADC) imaging obtained by the clinical stroke services at the time of each patient's admission to hospital was available to assist with determination of the boundary of regional infarction (see below). DWI and ADC images were performed in a Siemens Verio 3T scanner via diffusion‐weighted EPI sequences. Nineteen DWI axial slices were acquired at *b* = 0, 500 and 1,000 s/mm^2^ covering the entire brain. Other imaging parameters were: TR/TE 4,600/90 ms, field of view 267 × 267, matrix 192 × 192, slice thickness 5 mm, with 6.5 mm spacing. The ADC maps were acquired with identical parameters with *b* = 1,000 s/mm^2^ .

### Breath‐Hold Paradigm and End‐Tidal CO_2_ Pre‐Processing

Each participant performed a breath‐hold task during the BOLD imaging to measure vascular reactivity [Bright and Murphy, 2013; Murphy et al., 2011]. The task consisted of 14 s of natural breathing, followed by 16 s paced breathing, then a 15 s end‐expiration breath‐hold down to an unforced depth. After the breath‐hold, a quick exhalation of residual air was performed prior to a return to natural breathing, which allowed the measurement of end‐tidal CO_2_ increases as a result of the breath‐hold. This cycle was repeated six times over the course of the scan. The participants were trained outside the scanner and instructed to proceed to each stage by a series of visual pictorial and written cues presented at the centre of a screen.

End‐tidal CO_2_ traces were recorded throughout the experiment at a sampling frequency of 200 Hz, via a nasal cannula attached to a Medrad Veris capnograph. The recorded end‐tidal CO_2_ was converted to units of mmHg. A home‐grown peak‐detection algorithm was used to determine end‐tidal CO_2_ values by determining the partial pressure of CO_2_ at the end of each breath. A linear interpolation was made between the end‐tidal CO_2_ measure of the final breath before the breath‐hold and the quick exhalation CO_2_ measure after the breath‐hold. After this interpolation, a scale invariant convolution with a HRF was performed so that the scaling of the end‐tidal CO_2_ regressors remains the same. Convolution with a standard double gamma variate HRF [Friston et al., 1998] was performed because it has been demonstrated that more variance can be explained in the BOLD breath‐hold data [Murphy et al., 2011]. In this way, the amplitude of the regressors reflects the true increase in end‐tidal CO_2_ so that a quantitative measure of CVR could be made.

### fMRI Pre‐Processing

The BOLD breath‐hold data were corrected for motion using AFNI's *3dvolreg* program by performing a rigid body alignment of each volume to the middle volume (http://afni.nimh.nih.gov/afni/). Each voxel's time series was converted to a %BOLD signal time series by dividing by the mean across time points. A global %BOLD time series was calculated for each participant by averaging over all voxels in the brain. Linear drifts were removed from the data. No smoothing was performed to avoid contamination of CVR values between different regions.

### Analyses of Breath‐Hold Data—GlobOpt, VoxOpt and RHsig

With the aim of determining the best way to analyse the breath‐hold data, three analyses were performed: Global Optimization (*GlobOpt)*, Voxel‐wise Optimization (*VoxOpt)* and Right Hemisphere Signal (*RHsig*; Fig. 1c). First, to remove delays between the BOLD data and the end‐tidal CO_2_ trace that are caused both by physiological processes (such as the time for alveolar diffusion of CO_2_ in the lung, and the time for blood to travel between the lungs and brain) and by the experimental apparatus (delays along the long sampling line between the participant in the MR scanner and the capnograph), the delay was optimized to the corresponding global BOLD signal (see top panel in Fig. 1c). In practice, this was performed by temporally shifting the end‐tidal CO_2_ trace between −15 s and +15 s in 0.1 s steps, to find the delay that explained the most variance in the global BOLD signal. This globally optimized CO_2_ trace then formed the regressor for the *GlobOpt* analysis; that is, it was fitted to each voxel's time series with a general linear model (GLM) using AFNI's *3dDeconvolve* function. The beta weight from the regression for each voxel gives the breath‐hold response in that voxel in units of %BOLD signal change per mmHg change in end‐tidal CO_2_.

The *VoxOpt* analysis differed from the *GlobOpt* analysis by allowing the CO_2_ delay to be optimized on a voxel‐wise basis. In practice, a similar analysis was performed where the CO_2_ trace was included as a regressor in a GLM. This was repeated for each delay of the CO_2_ trace (−15 s to +15 s in 0.1 s steps from the globally optimized delay value). On a voxel‐wise basis, the delay at which the GLM explained the most variance in that voxel was chosen as the optimal delay. Again, the beta weight in that voxel for that delay gave the breath‐hold response in units of %BOLD signal change per mmHg change in end‐tidal CO_2_.

The final analysis, *RHsig*, provides a way of obtaining breath‐hold response maps if the CO_2_ trace is unavailable or of too poor a quality to use. If an area of healthy tissue can be defined, the average BOLD response across that region provides a good model of the expected breath‐hold response that can be included as a regressor in a GLM. In the *RHsig* analysis, it was assumed that the average response over the entire right hemisphere is a good representation of the BOLD signal response to the breath‐hold challenge. A similar approach was used by van Oers et al. [2010]. This average was fitted to each voxel in a GLM, and the derived beta weight for that voxel was the breath‐hold response in units of %BOLD (as a ratio of the %BOLD signal change of the *RHsig*, that is, 100% indicates a signal with the same amplitude as the *RHsig* time series).

### Lesion, Peri‐infarct and Healthy Tissue Masks

Individual 3D lesion masks were manually delineated by a neurologist (FG) and verified by a second neurologist (RJSW). These were drawn one slice at a time, on T_1_‐weighted images using *FSLView* (http://www.fmrib.ox.ac.uk/fsl/), with additional guidance from the diagnostic acute post‐stroke DWI and ADC sequences that were available in 41 subjects. DWI sequences reveal the early infarct with high contrast, and were used to guide the boundaries of the lesion on the high resolution T_1_‐weighted structural images [Saur et al., 2006; Mah et al., 2014]. Similar lesion identification based on T_1_‐weighted images is commonly used in functional neuroimaging studies that investigate recovery of cognitive functions after stroke, which can arguably be confounded by changes in CVR [Bates et al., 2003; Brownsett et al., 2014; Dronkers et al., 2004; Crinion et al., 2006, Teki et al., 2013, Saur et al., 2006]. Identification of the lesion manually, is considered gold standard when evaluating the efficacy of automated lesion segmentation algorithms [Mah et al., 2014; Seghier et al., 2008]. Session specific lesion masks were defined independently for each of the *SPPS* and *CPPS* scans.

Two patients had acute infarcts that included lesions (maximum volume 1.4 cm^3^) in the right hemisphere in addition to the acute infarct in the left hemisphere (Fig. 1a). These were included in the acute lesion mask. Five patients had an additional remote and small chronic lesion (range of lesion volume: 0.09–30.6 cm^3^) that was excluded from the acute lesion mask.

A schematic of the masks used in this study is shown in Figure 1b. The acute lesion mask was denoted *L‐Lesion* since the acute stroke lesions were confined to the left hemisphere in the majority of cases, and predominantly left‐lateralized in the few patients with bilateral acute strokes. A peri‐infarct tissue mask, *L‐PeriInfarct*, was defined for each patient by dilating the individual's *L‐Lesion* by 10 mm and then removing voxels that were in *L‐Lesion*. The remaining left hemisphere voxels outside *L‐Lesion* and *L‐PeriInfarct* constituted the *L‐Healthy* mask. Homologous masks were defined in the right hemisphere and were named *R‐Lesion*, *R‐PeriInfarct* and *R‐Healthy*. This was done by transforming the left hemisphere masks into Montreal Neurological Institute (MNI) standard space (see below), then flipping about the left/right plane, and finally transforming it back into individual subject space. *R‐Lesion*, *R‐PeriInfarct* and *R‐Healthy* contained unaffected tissue. Full left and right hemisphere masks comprising all voxels (including those in the lesion and peri‐infarct regions) in the hemisphere were also defined and called *L‐Hemi* and *R‐Hemi*, respectively. These were the only masks used for the *HV* group.

The high‐resolution structural images were registered to the MNI space using FMRIB's (http://www.fmrib.ox.ac.uk/fsl/) Linear Image Registration Tool (FLIRT) with 12 degrees of freedom and cost‐function masking to avoid the known problem of stretching normal tissue to fill the infarct during standard registration [Brett et al., 2001]. The final transformation matrix was used to transform each mask to standard space as stated above.

### Breath‐Hold Response Comparisons

**Figure 2:**
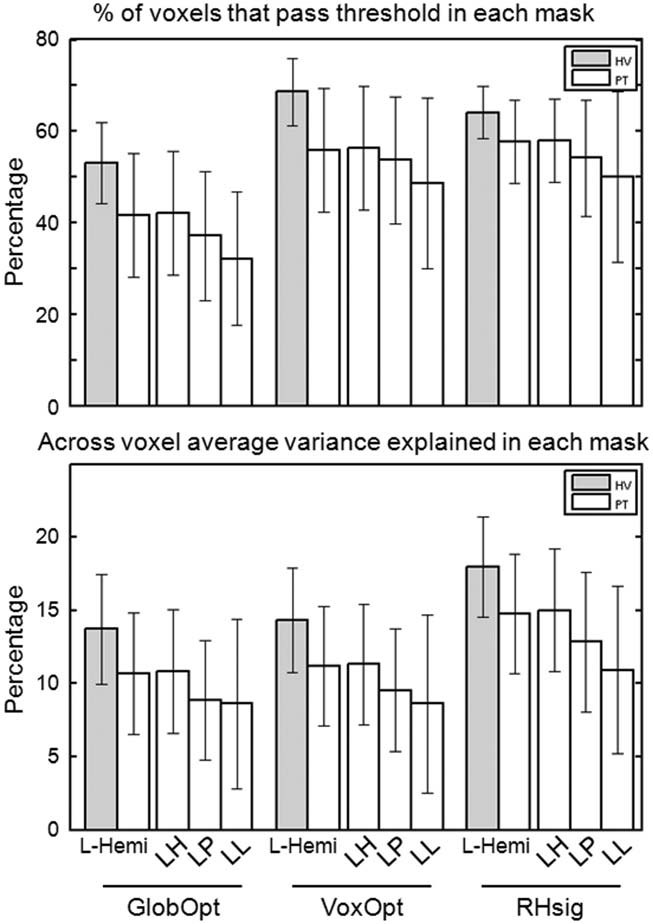
The three analyses methods (*GlobOpt*, *VoxOpt* and *RHsig*) were compared across different masks for *Sess1* (similar results were found for *Sess2*): the *L‐Hemi* mask for both the *HV* and PT groups and the *L‐Healthy* (LH), *L‐PeriInfarct* (LP) and *L‐Lesion* (LL) masks for the PT group. Grey bars depict the HV results and white bars the PT results (bars show means, error bars show standard deviations). The top panel shows the percentage of voxels in each mask that passed the liberal fitting threshold (*R^2^* = 0.0288, *P =* 0.05, no correction for multiple comparisons) averaged across subjects in each group. The bottom panel shows the across voxel average variance explained in each thresholded mask, averaged across the subjects in each group.

Breath‐hold responses for each of the three analyses were averaged over each mask for comparison. However, to remove voxels in which no breath‐hold response was observed (e.g., a white matter voxel in which blood volume is low), each mask was thresholded on the basis of both the individual participant and the analysis method. A liberal threshold of R^2^ = 0.03 (P = 0.05, for the number of time points with no correction for multiple comparisons) was used. If the GLM did not explain this amount of variance in a voxel's data, the voxel was removed from the mask. This ensured that averages across the masks were not skewed by voxels in areas of post‐stroke atrophy that may show no response. In each mask, both the percentage of voxels surviving this threshold and their average variance were calculated for all participants. Comparisons between analysis methods and across masks were made using paired and unpaired t‐tests. Specifically, differences in the values in each mask across the analysis methods were tested using paired t‐tests. Similarly, differences within analysis method but across the masks were tested using paired t‐tests. Unpaired t‐tests were used to test differences between the HV and PT groups in the L‐Hemi masks. These results are presented in Figure 2 and discussed below.

**Figure 3:**
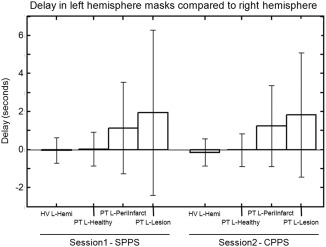
Using the *VoxOpt* analysis, the delay between each individual voxel time series and the end‐tidal CO_2_ time series can be calculated. With the assumption that the right hemisphere masks are largely unaffected by the stroke, the delay in the left hemisphere masks compared with their right homologous regions are shown for both the *SPPS* and *CPPS* time points (bars show means, error bars show standard deviations).

The average voxel‐wise delays across the masks, calculated using the VoxOpt analyses, were also compared for both the SPPS and CPPS time points. The average delay difference was calculated in the HV *L‐Hemi*, PT *L‐Healthy*, PT *L‐PeriInfarct* and PT *L‐Lesion* masks with the corresponding delay in the homologous mask as the baseline. In this way, no difference in delay between the right and left hemisphere yields a value of 0. T‐tests against 0 determined whether the delays were significantly different between the hemispheres. The results are shown in Figure 3 and discussed below.

**Figure 4:**
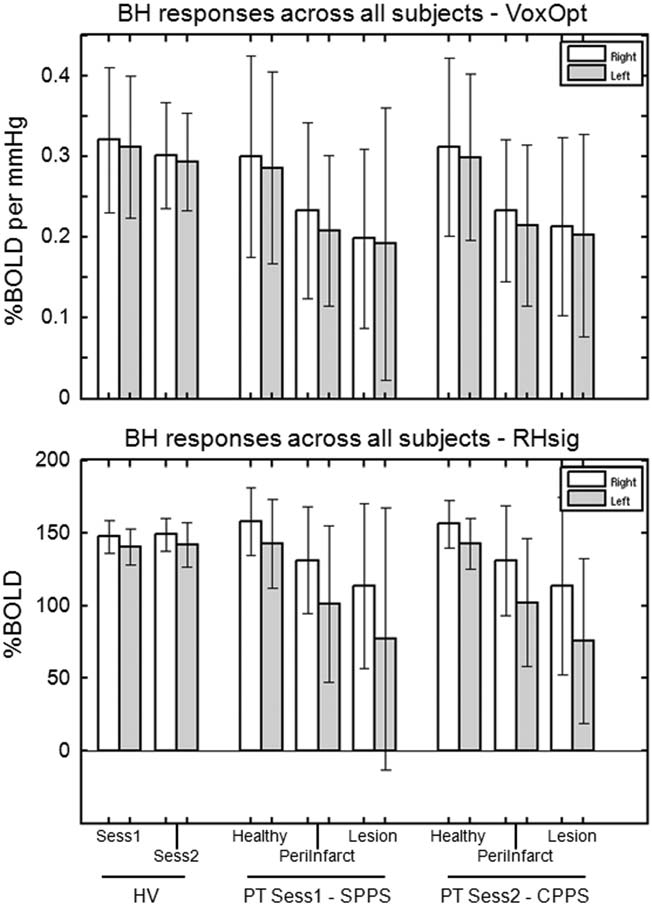
Breath‐hold responses averaged across participants (bars show means, error bars show standard deviations). The top panel displays results from the *VoxOpt* analysis and the bottom panel shows results from the *RHsig* analysis. White bars show results from masks in the right hemisphere and grey bars show results from the left hemisphere masks. For the HV group, whole hemisphere masks were used. For the *PT* group at the two time points (*Sess1*‐*SPPS* and *Sess2‐CPPS*), results from three masks in each hemisphere are shown: *Healthy*, *PeriInfarct* and *Lesion*.

CVR and %BOLD change responses were compared across left and right masks and *SPPS* and *CPPS* time points for both the *VoxOpt* and *RHsig* analyses, respectively. Paired t‐tests were used to determine significant differences between masks within analysis methods and between time points. Unpaired t‐tests were used to compare the PT and HV groups. The results are shown in Figure 4 and discussed below.

**Figure 5:**
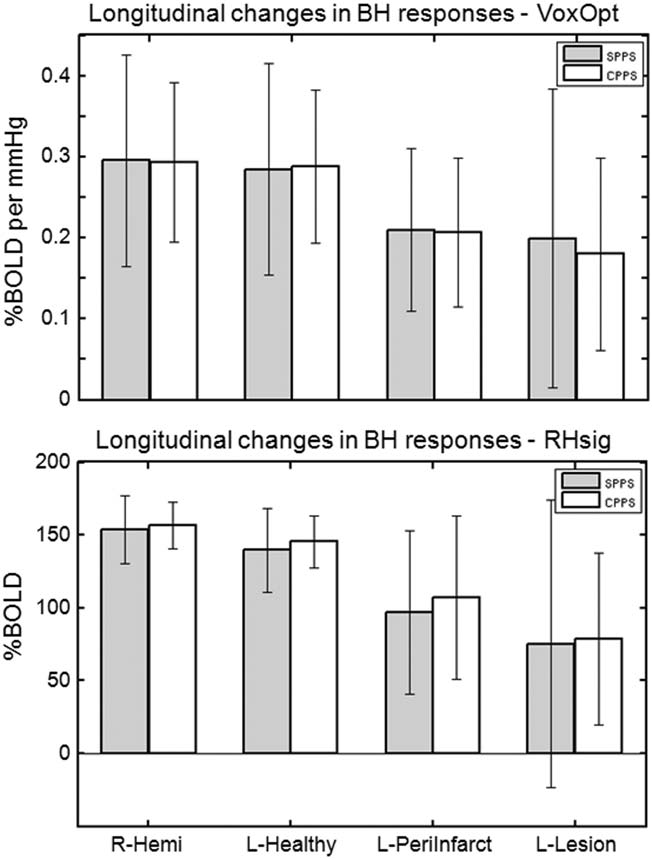
Breath‐hold responses averaged across patients with a scans at both *SPPS* and *CPPS* time points (*n* = 20; bars show means, error bars show standard deviations). The top panel displays results from the *VoxOpt* analysis and the bottom panel shows results from the *RHsig* analysis. The grey bars show results from the first *SPPS* session and the white bars from the second *CPPS* session. In both analyses, there were no significant differences between the *SPPS* and *CPPS* responses in each of the four masks; *R‐Hemi*, *L‐Healthy*, *L‐PeriInfarct* and *L‐Lesion*.

Finally, to determine whether the breath‐hold responses in the PT group change over time from *SPPS* to *CPPS*, the *Sess1* and *Sess2* results from the 20 patients that provided data for both phases were compared. Paired t‐tests were used to compare CVR results in each of the masks across the time points. These results are shown in Figure 5 and are discussed below.

## RESULTS

### Lesion Distributions

Stroke patients with recent left lateralized infarcts were chosen to take part in the study. Figure 1a shows the distribution of the lesions across the participants at each of the two phases, *SPPS* and *CPPS*. For the 24 patients scanned at *SPPS*, the mean percentage of left hemisphere voxels deemed to be in *L‐Lesion* was 1.6%, maximum 9%. *L‐PeriInfarct* comprised a mean of 7% of left hemisphere voxels, maximum 22%. In the 42 patients scanned at *CPPS*, *L‐Lesion* comprised a mean of 3.2% of left hemisphere voxels, maximum 21.2% and *L‐PeriInfarct* 10.8%, maximum 28.8%. In the 20 patients scanned at both *SPPS* and *CPPS*, the mean lesion size reduced from 1.78% to 1.28% of left hemisphere voxels, a small but significant difference (*t* = 3.2, *P =* 5 × 10^−3^).

### Motion Related to the Breath‐Hold Task

The average scan motion was calculated as the mean frame‐wise displacement (FWD) using the method described by Power et al. [2012]. Across both sessions, the mean FWD was 0.21 ± 0.13 in the PT group and 0.17 ± 0.08 in the HV group. An insignificant trend (*P* = 0.06) towards greater motion in the PT group versus the HV group was observed.

### End‐Tidal CO_2_ and the Global BOLD Signal

For the *GlobOpt* analysis, the delay at which the CO_2_ trace explained the most variance (as measure by *R^2^*) in the BOLD signal was chosen (see Fig. 1c top panel). The average *R^2^* did not differ in either the PT group or the HV group between the first and second time points (PT *Sess1* = 0.38 ± 0.28; PT *Sess2* = 0.38 ± 0.24; HV *Sess1* = 0.58 ± 0.21; HV *Sess2* = 0.47 ± 0.22). However, more global signal variance was explained by the CO_2_ trace in the HV group versus the PT group (*t =* 3.2, *P =* 2 × 10^−3^, combining the data across both sessions).

### Analysis Methods Comparison

Three types of breath‐hold analyses were compared: *GlobOpt*, *VoxOpt* and *RHsig*. Figure 2 shows the relative performance of each of the methods by plotting the percentage of voxels that passed threshold in each mask (top panel), and the average variance explained in each mask across the participants for *Sess1* (similar results were observed in *Sess2*). Results from the *HV* group are shown with grey bars. The percentage of voxels that passed threshold in left hemisphere masks, *L‐Hemi*, in this group was 53.1 ± 8.9% for the *GlobOpt* method, increasing to 68.6 ± 7.4% for the *VoxOpt* analysis and 64.1 ± 5.7% for the *RHsig* method. Both the *VoxOpt* and *RHsig* values were significantly greater than the *GlobOpt* values (*P <* 10^−7^ and *P =* 9 × 10^−5^, respectively). In the HV group, the variance explained in *L‐Hemi* voxels was 13.7 ± 3.7, 14.3 ± 3.6 and 17.9 ± 3.4% for the *GlobOpt*, *VoxOpt* and *RHsig* analysis methods, respectively. Again, both the *VoxOpt* and *RHsig* values were significantly greater than the *GlobOpt* values (*P <* 10^−7^ and *P =* 9 × 10^−6^, respectively) with the *RHsig* analysis also explaining significantly more variance than the *VoxOpt* method (*P =* 6 × 10^−5^).

Similar trends were observed in left hemisphere mask, *L‐Hemi*, of the PT group, with significantly less voxels passing threshold and significantly less variance explained with the *GlobOpt* analysis than both the *VoxOpt* and the *RHsig* analyses methods (% voxels: *P <* 10^−7^ and *P =* 2.8 × 10^−5^, respectively; variance explained: *P =* 1.4 × 10^−3^ and *P =* 1.2 × 10^−5^, respectively). However, in all three analyses methods, the results were lower in *L‐Hemi* in the *PT* group compared the HV group. Less voxels passed threshold in *L‐Hemi* of the PT group compared with the HV group (*GlobOpt*, *P =* 2 × 10^−3^; *VoxOpt*, *P =* 4 × 10^−4^; and *RHSig*, *P =* 9 × 10^−3^). A trend was observed for more variance explained in the HV compared with the PT group for the *GlobOpt* analysis (*P =* 0.014), with significantly more variance explained in both the *VoxOpt* and *RHsig* analyses (*P =* 8.6 × 10^−3^ and *P =* 7.2 × 10^−3^, respectively). A similar comparison between PT and HV in the *R‐Hemi* mask, in which areas should be unaffected, also shows decreases. Significantly less voxels pass threshold in the PT group for *GlobOpt* and *VoxOpt* analyses (*P =* 9.4 × 10^−3^ and *P =* 2.0 × 10^−3^). A trend for a reduction in the amount of variance explained in the PT is observed in the *R‐Hemi* masks for all analyses methods, with *VoxOpt* reaching significance (*P =* 3.5 × 10^−2^). This suggests that voxel time series are noisier in the PT group than the HV group.

In the PT group, *L‐Hemi* included not only tissue not directly affected by ischemic damage but also the lesion and the peri‐infarct masks. Since this may have caused the lower values in the PT group compared with the HV group, *L‐Healthy*, *L‐PeriInfarct* and *L‐Lesion* were also compared in Figure 2. For all three analyses, the number of voxels that passed the threshold in each mask, and the variance explained, reduced from *L‐Healthy* to *L‐PeriInfarct* and then to *L‐Lesion*. Only in the *GlobOpt* analysis was the reduction in the number of voxels significant (*L‐Healthy* vs. *L‐PeriInfarct, P =* 4.7 × 10^−4^; *L‐Healthy* vs. *L‐Lesion*, *P =* 1.7 × 10^−4^). For the measure of explained variance, all analyses showed significant reduction from *L‐Healthy* to the other two masks. For example, in the *VoxOpt* analysis, the variance explained in *L‐Healthy* was 11.3 ± 4.1%, which reduced significantly to 9.5 ± 4.1% in *L‐PeriInfarct* (*P =* 1.7 × 10^−3^) and to 8.6 ± 6.1% in *L‐Lesion* (*P =* 3.3 × 10^−3^).

### Delays in Breath‐Hold Responses Relative to the Contralesional Hemisphere

The *VoxOpt* analysis allowed for a variable delay between the CO_2_ trace and each voxel's time series, independent of other voxels. Figure 3 shows delays in the left hemisphere masks compared with their homologous mask in the right hemisphere. No difference in delays is defined as 0 on this graph. In the HV group, who were also scanned at two time points, the relative delay in *L‐Hemi* was −0.03 ± 0.67 and −0.15 ± 0.71 s for the *Sess1* and *Sess2*, respectively. Neither was significantly different from zero. Similarly, the response in *L‐Healthy* in the PT group was not significantly delayed relative to the homologous right hemisphere region, with mean delays of 0.03 ± 0.89 and −0.02 ± 0.87 s for the *Sess1‐SPPS* and *Sess2‐CPPS* data, respectively. The responses in *L‐PeriInfarct* and *L‐Lesion* showed a significant delay compared with their homologous right hemisphere regions at *Sess1‐SPPS*, with delays of 1.14 ± 2.4 s (*P =* 0.03) and 1.94 ± 4.34 s (*P =* 0.04), respectively. Likewise at *Sess2‐CPPS*, the responses in *L‐PeriInfarct* and *L‐Lesion* were also significantly delayed compared with their right homologues with delays of 1.24 ± 2.14 s (*P =* 5 × 10^−4^) and 1.83 ± 3.27 s (*P =* 7 × 10^−4^), respectively. This finding demonstrates the need to account for regional variability in delays to the CO_2_ trace in stroke‐affected areas during breath‐hold analyses. These delays do not appear to recover over the time periods tested in this study.

### Breath‐Hold Responses Across all Participants

The average breath‐hold responses across all participants are displayed in Figure 4 for the *VoxOpt* (top panel) and *RHsig* (bottom panel) analyses. For the *HV* group, values are shown in *L‐Hemi* and *R‐Hemi* for both *Sess1* and *Sess2*. For the *PT* group, both *Sess1‐SPPS* and *Sess2‐CPPS*, the results of the ipsilesional mask are displayed in grey bars (*L‐Healthy*, *L‐PeriInfarct* and *L‐Lesion*) and results from the right hemisphere are depicted in white bars (*R‐Healthy*, *R‐PeriInfarct* and *R‐Lesion*).

In the HV group, similar breath‐hold response levels were observed in both hemispheres and across sessions. For the *VoxOpt* analysis, *Sess1* showed mean responses of 0.32 ± 0.09 and 0.31 ± 0.09, and the corresponding values for *Sess2* were 0.30 ± 0.07 and 0.29 ± 0.06, for *R‐Hemi* and *L‐Hemi*, respectively (units: %BOLD per mmHg change in end‐tidal CO_2_). For the *RHsig* analysis, the average responses were 147 ± 11 and 141 ± 12 for *Sess1* and 149 ± 12 and 142 ± 15 for *Sess2* (units: %BOLD). Although the differences were small between hemispheres, they were significant for the *RHsig* analysis in both *Sess1* (*P =* 2.1 × 10^−4^) and *Sess2* (*P =* 9.6 × 10^−3^). This might be expected, because the regressor for the *RHsig* analysis was drawn from *R‐Hemi*, and therefore, was a slightly better fit to the voxels in that hemisphere. However, a similar significant difference was also observed for the *VoxOpt* analysis in both *Sess1* (*P =* 5.9 × 10^−3^) and *Sess2* (*P =* 0.023).

In the PT group, similar levels of response compared with the HV group were observed in the healthy tissue masks. For the *VoxOpt* analysis, the response in *L‐Healthy* was 0.29 ± 0.12 and 0.30 ± 0.10 for *Sess1* and *Sess2*, respectively (%BOLD per mmHg). The corresponding results for the *RHsig* analysis were 143 ± 30 and 143 ± 17 (%BOLD). For the two methods, there was a drop in response when going from *Healthy* to *PeriInfarct* to *Lesion* in both the stroke‐affected left hemisphere masks as well as the contralesional right hemisphere masks. For example, at *SPPS* with the *VoxOpt* method, the response dropped from 0.29 ± 0.12 to 0.21 ± 0.09 (*P* = 4 × 10^−5^) and to 0.19 ± 0.17 (although not significantly, *P* = 0.1) in *L‐Healthy*, *L‐PeriInfarct* and *L‐Lesion*. Similar declines from 0.30 ± 0.12 to 0.23 ± 0.10 (*P* = 5.3 × 10^−4^) and then to 0.19 ± 0.11 (*P* = 2 × 10^−3^) were also observed in the right hemisphere. This demonstrated that the definition of the masks changed the response levels on the supposedly unaffected right hemisphere side.

In the comparison between left and right hemisphere masks, the *RHsig* method appears to have distinguished the affected ipsilesional from the unaffected contralesional masks better, because the observed decline in response was greater on the left side (assuming that we would expect a decline in CVR on the lesioned side). For example, at *CPPS* the response difference was 156 ± 16 in *R‐Healthy* compared with 113 ± 61 in *R‐Lesion*, but on the affected side the difference was much greater, 142 ± 17 compared with 75 ± 56. Although, the left/right differences in the *VoxOpt* analysis appear small, paired *t*‐tests showed differences in *Healthy* (*Sess1*, *P =* 1.1 × 10^−3^; *Sess2*, *P =* 8.3 × 10^−4^) and *PeriInfarct* (*Sess1*, *P =* 7.8 × 10^−4^; *Sess2*: *P =* 9.5 × 10^−4^) but not *Lesion* in both sessions. The same comparisons in the three masks for the *RHsig* analysis showed more significant differences: *P =* 6.4 × 10^−4^, *P =* 4 × 10^−4^ and *P =* 0.015 for *Sess1‐SPPS*; *P <* 10^−7^, *P <* 10^−7^ and *P <* 3.6 × 10^−3^ for *Sess2‐CPPS*, respectively.

### Longitudinal Changes in Breath‐Hold Responses in 20 Patients with Two Scans

To determine whether reduced CVR recovers over time, *Sess1‐SPPS* and *Sess2‐CPPS* were compared in the 20 patients who were scanned twice (Fig. 5). For the *VoxOpt* analysis method (top panel) at the *SPPS* time point (grey bars), there was no significant difference between the mean responses in the *R‐Hemi* and *L‐Healthy* masks (0.29 ± 0.13 and 0.28 ± 0.13, respectively). The breath‐hold responses were significantly reduced to 0.20 ± 0.10 in the *L‐PeriInfarct* mask (*P =* 6.7 × 10^−4^). Although the *L‐PeriInfarct* had a similar response to the *L‐Lesion* mask (0.19 ± 0.18), due to a large standard deviation of the *L‐Lesion* responses, the difference between the *L‐Lesion* and *L‐Healthy* was not significant (*P =* 0.093). Almost identical values were observed for all four masks at *CPPS* (white bars). Importantly there was no significant difference between *SPPS* and *CPPS* in all the masks (*P >* 0.5).

Using the *RHsig* analysis (bottom panel of Fig. 5) at the *SPPS* time point (grey bars), the breath‐hold response in the *L‐Healthy* mask was significantly less than that in *R‐Hemi* mask (134 ± 29 and 153 ± 23, respectively: *P =* 2.8 × 10^−3^). Again, since the *RHsig* draws its regressor from the right hemisphere, this may not be surprising. There was a significant reduction from the *L‐Healthy* response to 96±56 in the *L‐PeriInfarct* mask (*P =* 8.1 × 10^−4^) and to 75 ± 99 in the *L‐Lesion* mask (*P =* 6.5 × 10^−3^). As with the *VoxOpt* analysis, no differences existed between responses in the *SPPS* and *CPPS* time points (*P >* 0.2), demonstrating that reductions in the breath‐hold response in the *Lesion* and *PeriInfarct* masks did not recover over the time course of this study.

### Results Summary

The *VoxOpt* and *RHsig* analyses fit the breath‐hold data better than the *GlobOpt* analysis (Fig. 2). Delays in the breath‐hold response in the *L‐PeriInfarct* and *L‐Lesion* masks are observed in the *PT* group at both the *SPPS* and *CPPS* time points (Fig. 3). At both time points, the CVR measures decrease from the *Healthy* to the *PeriInfarct* to the *Lesion* regions (Fig. 4). The decreased CVR in the *L‐PeriInfarct* and *L‐Lesion* masks does not recover over time (Fig. 5).

## DISCUSSION

Erroneous interpretation of BOLD fMRI group results can be caused by stroke‐induced changes in CVR. Bright and Murphy [2013] have previously established that CVR can be reliably quantified with breath‐hold tasks, even when poorly performed, demonstrating that this approach is useful in patients who may not comply fully with the task. Since stroke has a vascular etiology, changes to CVR, especially within the infarct and the peri‐infarct tissue, might be expected, making this a pathology for which this simple measure of CVR may be well used.

In this study, patients with left hemisphere infarcts were examined. In the sub‐group of patients that were scanned twice, *SPPS* and *CPPS* time points were approximately 4 months apart. A steady state of recovery is assumed at the *CPPS* time point since it has been shown that clinically, maximum recovery has been reached after 3 months, beyond which the rate of recovery plateaus and very little further recovery occurs [Laska et al., 2001; Pedersen et al., 1995; Ward et al., 2003].

In all patients, masks of four separate regions were defined: the lesion itself (*L‐Lesion*); the peri‐infarct tissue (*L‐PeriInfarct*); the regions in the left hemisphere unaffected by stroke (*L‐Healthy*) and the unaffected contralesional right hemisphere (*R‐Hemi*). The peri‐infarct tissue was arbitrarily defined as a 10‐mm wide region around the lesion. This mask was included in the analysis as some studies have attributed activity or lack of activity, in the peri‐infarct tissue to stroke recovery [Heiss et al., 1999, 2006; Rosen et al., 2000; Saur, 2006; Ward et al., 2003]. Although this mask was arbitrarily defined and may not fully overlap with affected areas, it was included as an example of a region that might have reduced CVR. In a BOLD study investigating recovery after stroke, any other region could be analysed in a similar way to determine if CVR changes are likely to cause interpretability problems for that region.

One aspect of the lesion and peri‐infarct BOLD data that might change compared with healthy tissue is the response delay to the breath‐hold. Previously, studies have used the end‐tidal CO_2_ trace as a regressor in a GLM [Bright and Murphy, 2013; Murphy et al., 2011] to produce a CVR measure that is comparable across subjects. If a region has a delayed response that is unaccounted for, the CVR measure might be artificially reduced. For this reason, we performed two methods of analysis with the CO_2_ trace: *GlobOpt* and *VoxOpt*. The *GlobOpt* method represents the standard analysis for breath‐hold data in which the same regressor is used for all voxels. The *VoxOpt* analysis allows for a voxel‐wise delay. Figure 3 demonstrates that the delay between the responses within the peri‐infarct and lesion tissue response compared with the responses in their homologous regions on the contralateral side can be appreciable [1.24 ± 2.14 s (*P =* 5 × 10^−4^) and 1.83 ± 3.27s (*P =* 7 × 10^−4^), respectively]. This can explain why less voxels passed threshold in *GlobOpt* analysis versus the *VoxOpt* analysis (Fig. 2). For this reason, the *VoxOpt* analysis was considered superior to the *GlobOpt* analysis, which was dropped from further study.

The *VoxOpt* analysis requires an end‐tidal CO_2_ trace, which may not be available in all situations. In addition, patients may have difficulties using the nasal cannula, especially if they prefer to breathe through the mouth, or the equipment may not be available in all settings. The finding that more global signal variance is explained by the end‐tidal CO_2_ trace in the HV group versus the PT group suggests that the end‐tidal CO_2_ recordings in the patient group may be less reliable. For this reason, the *RHsig* analysis, in which the model of breath‐hold signal change is derived from the contralesional hemisphere, was also compared. A similar number of voxels passed threshold with this method compared with the *VoxOpt* method. However, the *RHsig* method also explained significantly more variance in those voxels that passed threshold (Fig. 2). Perhaps this is not so surprising since the regressor derived from the *RHsig* is more likely to resemble a BOLD response than an end‐tidal CO_2_ trace convolved with a standard HRF. The advantages of the *RHsig* method are that it is a better fit to the individual voxel BOLD responses, and it does not need end‐tidal CO_2_ traces that require equipment and setup time that may not result in accurate recordings. Conversely, there are a number of disadvantages to the *RHsig* compared with the *VoxOpt* technique: it may not be truly comparable across subjects, since it is not a quantitative measure and, therefore, not suitable for correcting task‐related BOLD responses; it requires an assumption of where unaffected regions lie (indeed, in this study, seven of the *R‐Hemi* masks were slightly contaminated by small lesions in the right hemisphere); it does not account for delays between the model and a voxel's BOLD response; the model is an average over voxels that may have differing delays, and will, therefore, represent a more dispersed HRF; and finally, the CVR measures are biased towards the mask from which the regressor is derived, in this case *R‐Hemi*, and so may not be reliable across the entire brain.

Since it was difficult to determine which analysis approach was superior, results from both *VoxOpt* and *RHsig* analyses were investigated further. Comparisons of the responses in healthy unaffected tissue in the *PT* group with responses in the *HV* group showed no differences (Fig. 4). Although the number of voxels passing threshold is less, this suggests the CVR measures are as reliable in the patient group as the healthy controls. In both analyses, the CVR measure reduces as the mask size decreases (from *Healthy* to *PeriInfarct* and then *Lesion*) on both the stroke‐affected left hemisphere and the contralesional right hemisphere. It is possible that this represents a reduction in CVR due to the anatomical location of the masks. However, since the lesions are in largely heterogeneous locations (Fig. 1), it is more likely that this reduction is simply a function of mask size.

The interesting question is how the masks on the left side compare with their homologous masks on the right. A small but significant difference in CVR between the healthy tissue masks of the right and left hemisphere for the PT group might be indicative of increased cerebrovascular disease on the stroke‐affected left side of the brain. However, since a similar small significant difference is observed between the *L‐Hemi* and *R‐Hemi* mask in the HV group, it is difficult to determine whether this is the case. For the peri‐infarct mask compared with its homologue, small but significant differences were observed with the *VoxOpt* analysis, and much larger and significant differences observed with the *RHsig* analysis. Only the *RHsig* analysis showed differences in CVR between the lesion and its homologue (Fig. 4). If we expect that CVR is reduced in the lesion, this might suggest that the *RHsig* is better at distinguishing CVR changes in that area. However, from Figure 3, we know that there was a delay in the BOLD response in the lesion that was not accounted for by the *RHsig* analysis, which might have lead to the reduced CVR measures in the lesion compared with its homologue. From Figure 2, we can observe that the number of voxels that passed threshold was reduced in the lesion but it is possible that these voxels had intact CVR, albeit delayed, which the *VoxOpt* analysis captured but the *RHsig* method could not. Although differences exist, both methods provide similar interpretations of the data.

It is important to note that a breath‐hold challenge results in mild hypoxia alongside the intended hypercapnia. Since oxygen is vasoactive, this might directly influence the BOLD contrast [Bulte et al., 2012]. A previous study has shown that breath‐hold induced changes in end‐tidal O_2_ and CO_2_ traces are significantly negatively correlated [Bright and Murphy, 2013], thus their respective effects on BOLD signal changes are difficult to disentangle. However, it has been shown that there is minimal impact on CBF when end‐tidal O_2_ ranges from 60 to 150 mmHg [Brugniaux et al., 2007]; all changes to the breath‐hold task fall well within this range. A recent study comparing CVR measures derived from breath‐hold paradigms and gas challenges demonstrated that mild hypoxia caused by breath‐holds did not significantly alter CVR results compared with an iso‐oxic CO_2_ challenge [Tancredi and Hoge, 2013]. These studies indicate that the mild hypoxia caused by the breath‐hold challenge should not influence the CVR values in this study.

The peri‐infarct region is important when studying stroke recovery. Many longitudinal BOLD fMRI studies focus on activity in the peri‐infact tissue, to determine if neural activity has returned to what would be expected in healthy brains [Saur, 2006; Ward et al., 2003]. Others have attributed activity in the peri‐infarct region, or lack of it, to stroke recovery [Heiss et al., 1999, 2006; Rosen et al., 2000]. Alterations in the peri‐infarct CVR over time, may adversely affect the interpretation of task‐related BOLD signal change in fMRI studies of patients in the acute stages of stroke or in longitudinal studies. In this study, for both the *VoxOpt* and *RHsig* analysis methods, the CVR in the peri‐infarct tissue was shown to be reduced compared with the healthy tissue in the stroke‐affected hemisphere, but did not change between the sub‐acute and chronic phases after the ictus (Fig. 5). These findings suggests that in the cohort of patients studied, a relative lack of group‐level task‐related BOLD signal in the peri‐infarct tissue compared with the healthy controls, may not accurately reflect neural deficits. However, since CVR in the peri‐infarct tissue remains unchanged over time, a finding of increased group‐level peri‐infarct activity in the chronic phase in such a longitudinal study is less likely to be due to changes in vascular reactivity, and may be more reliably interpreted as a change in neural activity in the peri‐infarct tissue after stroke.

## CONCLUSIONS

This study demonstrates that CVR can be measured successfully in a stroke patient population using a breath‐hold task. The resulting CVR measures can be used to disentangle vascular and neural changes caused by stroke, increasing confidence when interpreting group‐level BOLD signal results in terms of neural activity (oxygen consumption). A lack of CVR differences in healthy tissue between patients and controls in this cohort suggests that task‐related BOLD signal changes in these regions should be unaffected by the stroke and be more representative of task‐related oxygen metabolism. In contrast, since CVR is reduced in the stroke peri‐infarct tissue, a lack of BOLD signal in that area compared with controls may not accurately reflect neural deficits. However, the CVR in the peri‐infarct tissue remains unchanged over time, suggesting that any group‐level BOLD increases in this region over time in a longitudinal fMRI study are less likely to be due to an improvement in vascular reactivity, and may be more confidently attributed to changes oxygen metabolism caused by altered neural function. This study provides a framework for stroke researchers to account for CVR‐related confounds in BOLD fMRI stroke studies. By including a similar breath‐hold protocol in longitudinal studies of stroke recovery and performing similar analyses, researchers can more confidently interpret BOLD signal changes by disentangling vascular and neural influences using this method.
